# Effect of Glue Spreads on the Structural Properties of Laminated Veneer Lumber from Spindleless Rotary Veneers Recovered from Short Rotation *Hevea* Plantation Logs

**DOI:** 10.3390/polym13213799

**Published:** 2021-11-03

**Authors:** Pui San Khoo, Kit Ling Chin, Chuan Li Lee, Paik San H’ng, Mohd Sahfani Hafizuddin

**Affiliations:** 1 Institute of Tropical Forestry and Forest Products, Universiti Putra Malaysia, Serdang 43400, Malaysia; gs43501@student.upm.edy.my (P.S.K.); chuanli@upm.edu.my (C.L.L.); gs59458@student.upm.edu.my (M.S.H.); 2 Faculty of Forestry and Environment, Universiti Putra Malaysia, Serdang 43400, Malaysia

**Keywords:** laminated veneer lumber, small-diameter *Hevea* log, spindleless rotary veneers, glue spread rate, structural properties

## Abstract

Unproductive young rubber trees (15 years old) with smaller diameters (15 to 18 cm) compared to conventional rubber logs, harvested at the age of 25 years old, were selected for the production of laminated panels. Spindleless rotary veneer peeling was applied to logs from short-rotation rubber forest plantations to produce veneers for structural purposes. This raises questions about the utilization of these small-diameter logs with respect to its effect on the quality of veneer and laminated panels produced. This study examines the effect of the glue spread rates on the physical and mechanical properties of rubberwood laminated veneer lumber (LVL). Analysis of variance shows that the application of a 280 g/m^2^ glue spread rate significantly improved the density, water absorption and dimensional stability of rubberwood LVL. The mechanical properties of rubberwood LVL produced with a 200 g/m^2^ glue spread rate met the minimum requirement for the 2.1E-3100F stress class; 91.05 MPa for the modulus of rupture in the flatwise direction and 50.23 MPa for compressive strength parallel to the longitudinal axis. The modulus of elasticity in the flatwise direction of 11,189.55 MPa reached the minimum requirement for the 1.5E-2250F stress class.

## 1. Introduction

Due to dwindling replanting hectarage, the demand for rubber logs could soon outstrip supply. To ensure the supply of timber and latex, a higher planting density has been implemented in rubber forest plantations (RFPs). These RFPs have a 15-year cycle, as opposed to the conventional 25-year cycle. This is specifically for the exploitation of wood with the option of latex exploitation. Despite the fact that wood is the main RFP yield, latex production is still a primary concern to collect. In recent years, many rubber holders introduced young-tapping and high-frequency tapping systems in RFPs to increase latex yields. However, this has an impact on the growth of rubber trees over their life cycle, resulting in a slower increase in girth. Rubber trees are harvested at a younger age and have a smaller diameter (15 to 18 cm) under the RFP planting and modern tapping system than under the conventional planting and tapping technique used years ago. This raises concerns about the impact of using these small-diameter logs on the quality of the veneer and the laminated panels produced.

Peeling small-diameter logs resulted in veneers of different quality compared to peeling large-diameter logs of the same wood species, especially in terms of lathe check properties and wettability. Effective usage of glue is highly dependent on the characteristics of the veneer surface, with the roughness of the veneer being one of them. Rubberwood veneers peeled and recovered from short rotation *Hevea* plantation logs using spindleless lathe have deep lathe checks and rough surfaces, which might cause the adhesive to over-penetrate and dry out at the glueline [1]. Over-penetration of adhesive into wood results in insufficient adhesive remaining in the glueline to bridge between wood surfaces [2]. The presence of insufficient adhesive in the glueline decreased cohesion strength, resulting in a thin and starved glueline [3]. This means that the glueline cannot be formed, and hence there is no bonding strength between the veneers [4]. As a result, a higher glue spread rate is needed to compensate for the effects of high-penetration levels. Veneers with a deep and high frequency of lathe checks may require a higher glue spread rate to create an adequate glueline thickness [5,6], allowing better internal surface contact for mechanical interlocking, and transferring stresses efficiently between plies [4] in order to rework the defectives of the veneer surface topography.

The glue spread rate of veneer affects the strength properties of laminated panels. Moreover, increasing the application of glue does not always improve the bonding strength but even diminishes it. Vick [5] claimed that structural panels with a glueline thickness of 0.076 mm to 0.152 mm are able to withstand maximum mechanical loading stresses and dimensional changes. Below this range, the glueline is too thin to effectively transfer stresses from one adherent to the other, particularly stresses caused by moisture-induced dimensional changes [4,7]. When glueline thickness increased to a certain point, the strength gradually deteriorates [4,8] as thick gluelines are brittle and easily fracture [5]. Thus, the glueline thickness of laminated veneer lumber (LVL) should be within a certain range because it directly affects the panes strength [4].

The glue spread rate is one of the most important processing factors in achieving an optimal glueline thickness [4,9]. The adhesive has important technological and economic implications in terms of the use of wood products and production costs. The cost of the adhesive will account for up to half of the LVL cost [10]. By optimizing the glue spread rate, it is possible to avoid the use of excessive adhesive while maintaining a satisfactory LVL production cost and quality. Therefore, the aim of this study was to optimize the physical and mechanical properties of rubberwood LVL by means of the glue spread rate.

## 2. Materials and Methods

### 2.1. Fabrication of LVL

Small-diameter rubberwood logs (between 15 and 18 cm) from short-rotation *Hevea* plantations (Kuala Kangsar, Perak, Malaysia) were peeled using a spindleless rotary-peeler (HK-130; Linyi Hengkai Machinery Manufacture Factory, Shandong, China) according to the method demonstrated by Khoo et al. [1] to produce veneers with 2 mm thickness, and the properties are listed in Table 1.

**Figure 1:**
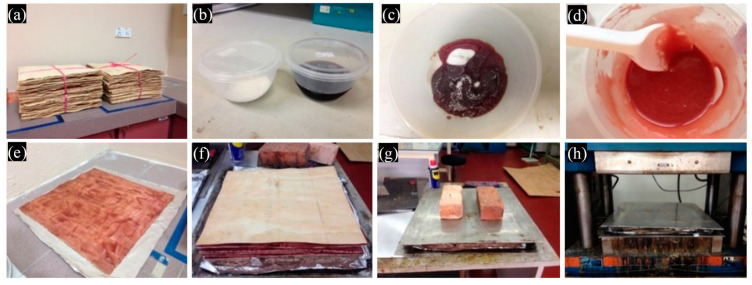
Fabrication of seven-ply rubberwood LVL; (**a**) veneer size: 400 × 400 × 2 mm (**b**) ratio of PF to filler is 3:1; (**c**) PF and filler were mixed thoroughly; (**d**) ready-use resin; (**e**) resin application on the veneer; (**f**) LVL lay-up with veneer tight side facing tight side and loose side facing loose side; (**g**) cold pressing for five minutes; and (**h**) hot pressing for twelve minutes.

**Figure 2:**
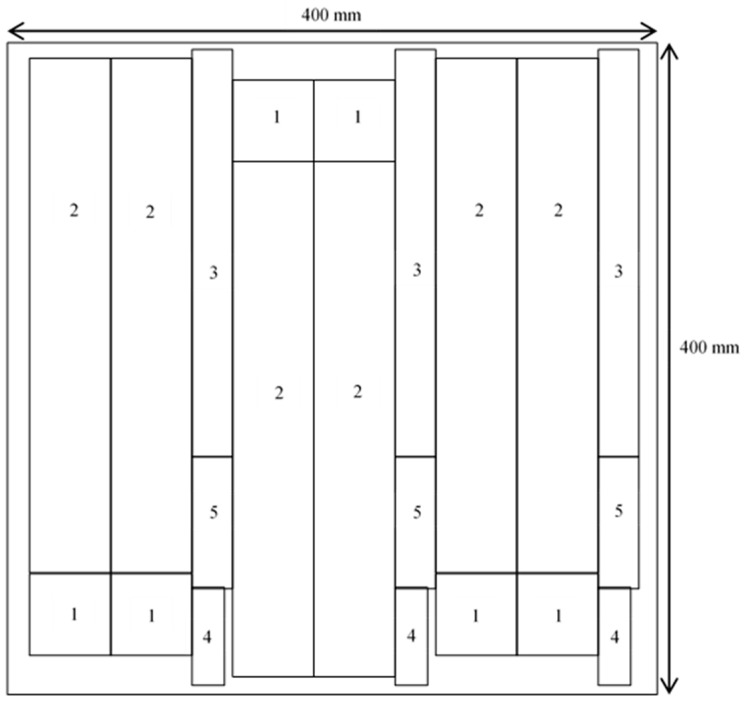
Cutting pattern of specimens for physical and mechanical tests. Note: (1) physical tests such as density, specific gravity, water absorption, radial swelling, longitudinal swelling, tangential swelling and volumetric swelling after two and 24 h; (2) static bending; (3) tensile parallel to the longitudinal axis; (4) compression parallel to the longitudinal axis; (5) gluebond shear test.

Rubberwood LVL was made from 2-mm-thick rotary veneers peeled from a spindleless lathe. Rubberwood veneers that had been rotary peeled were conditioned at a moisture content of 7% ± 1%. Figure 1 shows the fabrication process of seven-ply rubberwood LVL. Phenol formaldehyde (PF) adhesive with 45% solid content were provided by AICA Malaysia Sdn. Bhd. The following were some of the properties of the PF adhesive: specific gravity of 1.2 at 30 °C; pH of 12.0–14.5; viscosity of 60 to 110 Cp at 30 °C and gel time of 15 to 30 min at 105 °C. With the PF adhesive, a 1:3 ratio of commercial filler was added. On the veneer surface, glue spread rates of 200, 240, and 280 g/m^2^ were applied. With the loose side facing the loose side and the tight side facing the tight side, seven veneer sheets were pressed together parallel to each other. The LVL with 7 plies were cold pressed for 5 min and then hot pressed for 12 min at 120 °C with a specific pressing pressure of 9 kgf/cm^2^ [11]. After hot pressing, the LVLs were conditioned at a temperature of 20 °C ± 3 °C and a relative humidity of 65% ± 1% until they achieved an equilibrium moisture content. The LVLs were then cut into specimens to be used in the physical and mechanical tests. The sizes of specimens for various tests were depicted in Table 2. Figure 2 shows the LVL specimen cutting pattern for physical and mechanical tests.

### 2.2. Evaluation

#### 2.2.1. Determine the Moisture Content

The moisture content of the specimens was tested according to the ASTM D 4442-07 [12]. The specimen’s initial weight was recorded. The specimens were then oven-dried at 103 °C ± 2 °C until a constant weight achieved and weighed at an accuracy of ±0.01 g. Equation (1) was used to calculate the moisture content of the specimens.

(1) $$\mathrm{MC},\;\%=\frac{W_{i}-W_{o}}{W_{o}}\times 100$$

where,

$W_{i}$ = initial weight of the specimen

$W_{o}$ = oven-dry weight of the specimen

#### 2.2.2. Determine the Density

The density of LVL specimens measuring 50 mm by 50 mm was tested according to the ASTM D 2395-14 [13]. The specimens were air dried in the conditioning room to a moisture content of about 10% ± 2%. A weighing balance was used to weigh the mass of each LVL specimen. The volume of the specimens was measured by multiplying the specimen’s length, width, and thickness. Equation (2) was used to calculate the density of the specimens.

(2) $${\mathrm{Density}}_{12\%},\;\frac{g}{{\mathrm{cm}}^{3}}=\frac{\mathrm{Mass}}{\mathrm{Volume}}$$

where, 

Mass = mass of the specimen, g

Volume = volume of the specimen, cm^3^

#### 2.2.3. Determine the Specific Gravity

ASTM D 2395-14 [12] was used to determine the specific gravity of LVL specimens measuring 50 mm by 50 mm. The specimens were oven dried at 103 °C ± 2 °C until they reached a constant weight. A weighing balance was used to determine the mass of each specimen. The volume of the specimens was measured by multiplying the specimens’ length, width, and thickness. The weight and volume of oven-dried specimens were used to calculate oven-dry density. Equation (3) was used to calculate the specific gravity of LVL.

(3) $$\mathrm{Specific}\;\mathrm{gravity}=\frac{D_{0}}{D_{w}}$$

where

$D_{o}$ = density of wood when oven-dry

$D_{w}$ = density of water

#### 2.2.4. Determining the Water Absorption and Radial, Tangential, Longitudinal and Volumetric Swelling

LVL specimens measuring 50 mm by 50 mm were weighed and the radial, tangential and longitudinal lengths of the specimens were measured before being submerged horizontally in 25 mm of distilled water at a temperature of 20 °C ± 1 °C. The water was removed after two hours of submersion. The specimens were suspended to drain for 10 ± 2 min to remove excess surface water. The specimens were weighed and the radial, tangential and longitudinal lengths of the specimens were measured. The specimens were then submerged for an additional 22 h before undergoing the weighing and measuring process described above. After 24 h submersion, the specimens were placed in an oven at 103 °C ± 2 °C to determine the moisture content based on oven-dry weight. According to ASTM D 1037-12 [14], the absorbed water of the specimens was calculated as a percentage. Equations (4)–(6) were used to calculate the percentage of water absorption, as well as radial, tangential, longitudinal, and volumetric swelling.

(4) $$\mathrm{Water}\;\mathrm{absorption},\;\%=\frac{W_{f}-W_{i}}{W_{i}}\;\times \;100$$

(5) $$\mathrm{Swelling},\;\%=\frac{L_{f}-L_{i}}{L_{i}}\;\times \;100$$

(6) $$\mathrm{Volumetric}\;\mathrm{Swelling},\;\%=\frac{V_{f}-V_{i}}{V_{i}}\times 100$$

where

$W_{f}$ = final weight of the specimen after being immersed in water

$W_{i}$ = initial weight of the specimen before being immersed in water

$L_{f}$ = final length of the specimen after being immersed in water

$L_{i}$ = initial length of the specimen before being immersed in water

$V_{f}$ = final volume of the specimen after being immersed in water

$V_{i}$ = initial volume of the specimen before being immersed in water

#### 2.2.5. Determining the Modulus of Rupture (MOR) and Modulus of Elasticity (MOE)

Flatwise three-point static bending tests were performed on specimens with dimensions of 12 mm thickness by 50 mm width by 316 mm length using an INSTRON Universal Testing Machine in accordance with ASTM D 5456-10 [15]. The span-to-depth ratio was 18 and speciments with a depth-to-width ratio of three or greater needed to be laterally supported with lateral supports. Throughout the test, the crosshead loading speed was maintained at 1.05 mm/min. Equations (7) and (8) were used to calculate the MOR and MOE of each specimen.

(7) $$\mathrm{MOR}\;\left(\mathrm{MPa}\right)=\frac{3\mathrm{PL}}{2{\mathrm{bd}}^{2}}$$

(8) $$\mathrm{MOE}\;\left(\mathrm{MPa}\right)=\frac{P_{1}L^{3}}{4{\mathrm{bd}}^{3}Y_{1}}$$

where 

P = maximum load, in Newtons;

P_1_ = load at proportional limit, in Newtons;

Y_1_ = center deflection at proportional limit load, in mm;

L = length of span, in mm;

b = width of the specimen, in mm;

d = thickness of the specimen, in mm.

#### 2.2.6. Determining the Compressive Strength Parallel to the Longitudinal Axis

According to ASTM D 5456-10 [15], the specimen size for compression parallel to the grain was 12 mm by 20 mm by 60 mm. The loading speed of the crosshead was kept constant at 0.06 mm/min. Equation (9) was used to calculate the compressive strength parallel to the longitudinal axis.

(9) $$\mathrm{Compressive}\;\mathrm{strength}\;\left(\mathrm{MPa}\right)=\frac{P}{\mathrm{bd}\;}$$

where

P = maximum load

b = width of the specimen, in mm;

d = thickness of the specimen, in mm.

#### 2.2.7. Determining the Tensile Strength Parallel to the Longitudinal Axis

LVL specimens with dimensions of 12 mm thickness by 25 mm width by 250 mm length for tension parallel to the longitudinal axis were tested according to ASTM D 5456-10 [15]. The loading speed of the crosshead was maintained at 0.15 mm/min. Equation (10) was used to calculate each specimen’s tensile strength parallel to the longitudinal axis.

(10) $$\mathrm{Tensile}\;\mathrm{strength}\;\left(\mathrm{MPa}\right)=\frac{P}{\mathrm{bd}}$$

where

P = maximum load

b = width of the specimen, in mm;

d = thickness of the specimen, in mm.

#### 2.2.8. Determining the Gluebond Shear Strength

According to ASTM D 906-11 [16], the specimen size for gluebond shear testing was 81 mm length by 25 mm width by 12 mm thickness. Two 3-mm grooves were cut to a depth of three and four plies for each side, and the glue shearing area was kept at 25 mm by 25 mm. The crosshead loading speed was maintained at 4 mm/min throughout the test. Equation (11) was used to calculate the gluebond shear strength of each specimen.

(11) $$\mathrm{Gluebond}\;\mathrm{shear}\;\mathrm{strength}\;\left(\mathrm{MPa}\right)=\frac{F}{l\;\times \;b}$$

where

F = failing force of the specimen

l = length of the shear area

b = width of the shear area

#### 2.2.9. Scanning Electron Microscopy (SEM)

An EM-30AX scanning electron microscope (COXEM, Daejeon, Korea) with a 20 kV acceleration voltage was used to observe the adhesive penetration into the cell wall. For testing, 10-mm-wide by 10-mm-long by 12-mm-thick specimens were cut at the cross section of each LVL.

### 2.3. Statistical Analysis

Statistical analyses were carried out using the statistical package SPSS for Windows, version 16.0 (SPSS, Chicago, IL, USA). Analysis of variance (ANOVA) was used to evaluate the effect of the glue spread rate on the physical and mechanical properties of LVL rubberwood. The effects were considered not to be statistically significant when the *p*-value was higher than 0.05 at the 95% confidence level.

## 3. Results and Discussion

### 3.1. Morphological Properties

**Figure 3:**
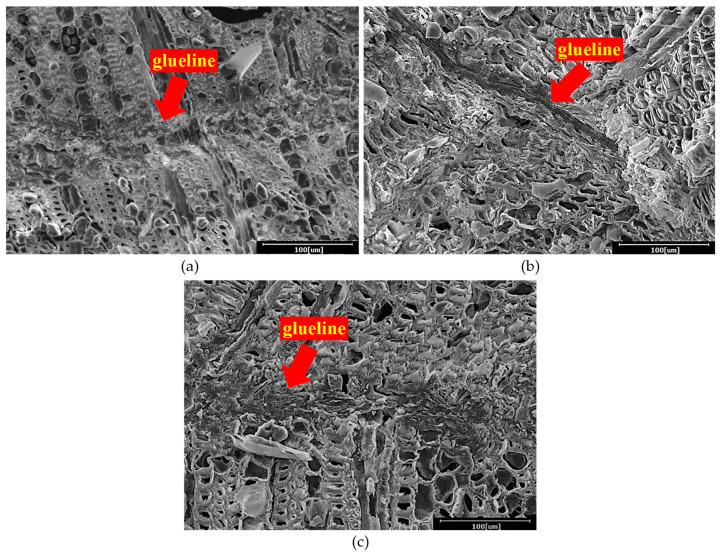
Glueline of rubberwood LVL observed using SEM with 500× magnification; LVL produced with (**a**) 200; (**b**) 240; and (**c**) 280 g/m^2^ glue spread rate.

Figure 3 presents cross sections of rubberwood LVL captured using SEM with 500× magnification. It could be observed that PF was able to penetrate into the wood structure for mechanical interlocking bonding. As a result, these would eventually allow more interaction between the phenol formaldehyde resin and veneers. However, the glueline thickness increased with the increasing of the glue spread rate (Figure 3). A more visible and thicker glueline was observed in the rubberwood LVL bonded with 240 and 280 g/m^2^ glue spread rates. Although thicker gluelines were observed, glue spread rates of 240 and 280 g/m^2^ for rubberwood veneers are considered excessive as excess adhesive was squeezed out around the edges of the panel during pressing. It is also known that with increasing glueline thickness, the bonding strength decreases; with a thicker glueline, higher internal stress is generated during glue shrinkage, which can lead to a lower shear strength [17].

**Figure 4:**
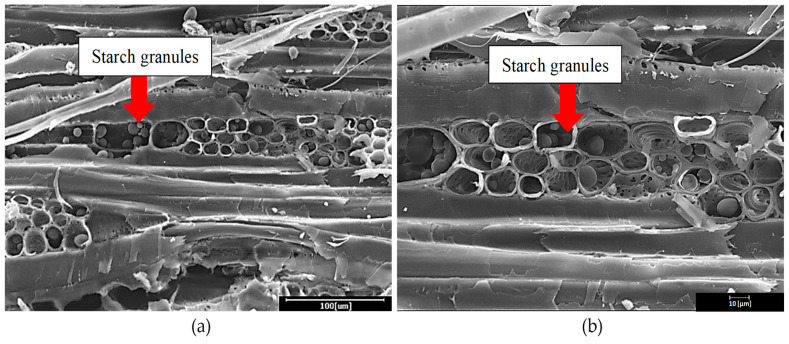
Starch granules stored in ray parenchyma cells of rubberwood (**a**) with 500× and (**b**) 1000× magnification.

Figure 4 shows the failure region of rubberwood LVL after a gluebond shear test, captured using SEM. These Figures present the starch granules deposited in the rubberwood ray parenchyma cells. Starch molecules are polymers of anhydrous glucose units formed and accumulated through the long-term energy storage of different plants in the form of unique and isolated granules [18,19]. According to Simatupang et al. [19], fresh rubberwood contains 1.05% to 2.29% free sugars and 7.53% to 10.17% starch. Due to the existence of large quantities of hydrophilic hydroxyl groups, starch is fitted with high hydrophilicity [20]. Starch molecules have strong intermolecular and intramolecular hydrogen bonds; therefore, they are hardly soluble at room temperature in water, and easily self-aggregate in organic media and form micron-scale aggregates in processing [21]. These characteristics of starch may impart poor compatibility with hydrophobic polymers [22]. Due to the hydrophobic nature of phenol formaldehyde resin [23], starch granules will prohibit the penetration of phenol formaldehyde and create a thick glueline formation if excessive adhesive is applied. With low adhesive penetration and the formation of a thick glueline, the veneers maybe unfavorable for gluing. Thick gluelines are undesirable because they reduce the mechanical strength of glued joints, causing the separation of adjacent layers [24,25].

### 3.2. Physical Properties

According to analysis of variance (ANOVA), the effect of the glue spread rate on all the physical properties of rubberwood LVL were highly significant (*p* < 0.01) except for moisture content. The average values that were significant were compared using Tukey’s test and are summarized in Table 3.

#### 3.2.1. Moisture Content, Density and Specific Gravity

After conditioning for two weeks, the average equilibrium moisture content was 12.68%, 12.91% and 13.13% for rubberwood LVLs produced from 2-mm-thick veneer using 200, 240 and 280 g/m^2^ glue spread rates, respectively. According to PRL-501 [26] and the Japanese Standard [27], the average moisture content of LVL intended for use in dry-service conditions should be less than 16% and 14%, respectively. The rubberwood LVL produced from this study meets the requirement of these standards.

Table 3 presents the average density and specific gravity of rubberwood LVL produced using a 2-mm-thick veneer and phenol formaldehyde at different glue spread rates of 200, 240 and 280 g/m^2^. Based on the verdicts, there was an increase in the density and specific gravity of rubberwood LVLs with the increasing of the glue spread rate. Rubberwood LVL produced with a 280 g/m^2^ glue spread rate has significantly higher density and specific gravity compared to 240 and 280 g/m^2^ glue spread rates (*p* ≤ 0.01). The density of phenol formaldehyde resin used in this study was 1.2 g/cm^3^, which was much higher than that of the rubberwood veneer. Therefore, the amount of phenol formaldehyde resin applied to the LVL has a greater effect on the total mass of the rubberwood LVL. The higher increase in the density and specific gravity of rubberwood LVL is due to a higher increment of the total mass of the panel [28,29,30].

#### 3.2.2. Water Absorption

As shown in Table 3, the effect of the glue spread rate on the water absorption percentage of rubberwood LVL was highly significant (*p* ≤ 0.01). Within the first two hours of immersion, rubberwood LVL produced with the highest glue spread rate, which was 280 g/m^2^, showed a significantly lower percentage of water absorption compared to the 200 and 240 g/m^2^ glue spread rates. After 24 h of immersion, the water absorption of rubberwood LVL produced using 240 and 280 g/m^2^ glue spread rates was significantly lower compared to that produced with the 200 g/m^2^ glue spread rate. A similar result was obtained by Abdul Khalil et al. [28], who reported that panels produced with a higher glue spread rate showed lower percentages of water absorption compared to the panels produced with a lower glue spread rates. Rubberwood LVL bonded with the highest glue spread rate has the lowest percentage of water absorption because more phenol formaldehyde resin penetrated into the wood cell wall, and chemically bonded to the hydroxyl group of wood structures [31]. Cross-linking reactions of phenol formaldehyde oligomer and -OH groups of cellulose occurred in the wood cell wall [32]. The formation of this cross-linked polymer reduced the numbers of free hydroxyl groups in the wood cell wall [31] and the wood cell wall became bulked with phenol formaldehyde oligomer [33]. During water immersion, the free hydroxyl group absorbs and forms hydrogen bond with water [34]; one of the scenarios for adhesive penetration into the cell wall is to occupy the free hydroxyl groups and reduce of the possibility of water absorption [3]. Thus, reducing the numbers of free hydroxyl groups weakens the diffusion of water molecules into the wood cell wall [31]. In other words, the compatibility between the hydrophilic fiber surface and the hydrophobic adhesive was higher when using higher spread rates [28]. A strong compatibility between the cellulose in the cell wall and the phenol formaldehyde oligomer could reduce the percentage of water absorption [23].

#### 3.2.3. Dimensional Stability

As shown in Table 3, rubberwood LVL bonded with a 280 g/m^2^ glue spread rate showed significantly (*p* ≤ 0.01) lower radial swelling after two- and 24-h of immersion, tangential swelling after two hours of immersion, longitudinal swelling after 24 h of immersion and volumetric swelling after 24 h of immersion. Rubberwood LVLs bonded with the highest glue spread rate were found to be the most dimensionally stable, with the least radial, tangential, longitudinal and volumetric swelling. This finding coincided with those of a previous study by Khalid et al. [35], who reported that a higher amount of adhesive improved the dimensional stability of the laminated compressed composite panels made from oil palm fronds. After hot pressing, internal stresses were built up in panels [36]. When the panels came in contact with water molecules, the water molecules penetrated and bonded with the free hydroxyl group in the cell wall through hydrogen bonding [37]. The internal stresses were released [36] and the cell structure attempted to restore to its initial form [38]. Thus, the interfacial bonding strength between the wood and the adhesive, as well as the cohesive forces in the wood and the adhesive have to be strong enough to counteract different swelling forces [36]. A higher glue spread rate ensures intimate contact between veneers [39]. Greater intimate contact eliminates surface voids and develops higher interfacial interaction between adhesives and veneers, further contributing to better dimensional stability of the panels [39,40].

Based on Table 3, the percentage of radial swelling was 2.30% ± 0.30% and 4.47% ± 0.47%, tangential swelling was 0.54% ± 0.03% and 1.25% ± 0.10%, longitudinal swelling was 0.10% ± 0.03% and 0.13% ± 0.03%, and volumetric swelling was 3.19% ± 0.32% and 6.08% ± 0.65%, after two- and 24-h immersion, respectively. Compared to the result in Khoo et al. [41], seven-ply rubberwood LVL was found to be more dimensionally stable compared to three-ply rubberwood LVL. The seven-ply rubberwood LVL has a significantly lower percentage of longitudinal swelling, tangential swelling and volumetric swelling, but a higher percentage of radial (thickness) swelling compared to the three-ply rubberwood LVL. A lower percentage of longitudinal, tangential, and volumetric swelling might be due to thicker LVL containing a higher amount of hydrophobic phenol formaldehyde resin, which blocks the pathway for water molecules to enter the wood cell wall when immersed in water. This was in agreement with the results Chai et al. [39], who stated that the glueline may have acted as a partially water-resistant layer that reduced the water intake of the porous material. For radial swelling, however, a thicker LVL containing more veneer plies may build up higher compression stresses in the panels during hot pressing compared to a LVL with less veneer plies. When panels are immersed in water, these compression stresses are released (spring back), particularly in the radial direction [36]. Higher compression stress resulted in severe spring-back and higher radial swelling.

### 3.3. Mechanical Properties

According to analysis of variance (ANOVA), the effect of the glue spread rate on MOR in the flatwise direction and compressive strength parallel to the longitudinal axis were highly significant (*p* < 0.01). There were no significant effects on the MOE in the flatwise direction, the tensile strength parallel to the longitudinal axis or the gluebond shear strength. The average values that were significant were compared using Tukey’s test and are summarized in Table 4.

#### 3.3.1. Modulus of Rupture (MOR) and Modulus of Elasticity (MOE)

**Figure 5:**
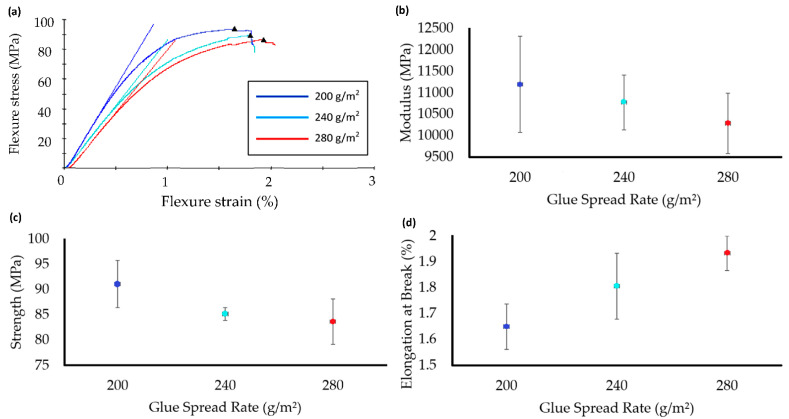
Mechanical characterization of LVL samples made with different glue spread rates by flatwise direction bending tests: (**a**) representative stress-strain curves for each treatment; (**b**) elastic modulus; (**c**) strength; (**d**) elongation at break.

Figure 5 shows the findings relating to the mechanical characterization of the LVL samples by bending testing in the flatwise direction. The stress-strain diagram based on the mean values shown in Figure 5a reveals different performance of the three LVL samples with different glue spread rates. The steeper curves of the LVL samples with lower glue consumption values indicate that the MOE decreased with the increase of the glue spread rate, which can also be seen in Figure 5b. The mean MOE in the flatwise direction, shown in Figure 5b, decreased by 3.8% and 8.1% for glue spread rates of 240 g/m^2^ (10,767.51 MPa) and 280 g/m^2^ (10,278.66 MPa) specimens, respectively, compared to 200 g/m^2^ (11,189.55 MPa) specimens. The mean bending strength in the flatwise direction, seen in Figure 5c, decreased by 6.5% and 8.2% for glue spread rates of 240 g/m^2^ (85.10 MPa) and 280 g/m^2^ (83.60 MPa) specimens, respectively, compared to the lowest glue consumption specimens of 200 g/m^2^ (91.05 MPa). Elongation at break, also known as fracture strain, is the ratio between the changed length and the initial length after breakage of the test specimen. It expresses the capability of the composite panel to resist changes of shape without crack formation. Higher glue consumption results in a slight increase in elongation at break but a decrease in the modulus of elasticity.

Based on the statistical analysis shown in Table 4, the application of a 200 g/m^2^ glue spread rate resulted in a significantly higher (*p* ≤ 0.01) MOR in the flatwise direction compared to 240 and 280 g/m^2^ glue spread rates. Excessive glue spread rates caused a reduction in the MOR of rubberwood LVL samples in the flatwise direction. In a study conducted by Khalid et al. [35], the MOR of oil palm frond composite panels was decreased due to excessive glue spread rates. The application of phenol formaldehyde resin is usually limited due to brittleness and the relatively high friction coefficient [42]. As a higher glue spread rate was applied, an excessive glueline was formed as a thick and brittle layer which resulted in a reduction in the MOR of the composite panel. On the other hand, a further increase in resin consumption led to a slight decrease in the MOE in the flatwise direction, but according to statistical analysis and homogeneous groups, these changes were not statistically significant (*p* > 0.05). Thus, the results presented a similar tendency as those of MOE in the flatwise direction, and were not affected by the glue spread rate. LVL manufactured with glue spread rates of 200 g/m^2^ had equally good MOE values as panels with an adhesive application of 240 and 280 g/m^2^. This may be related to their close densities and the fiber length of the small-diameter rubberwood used in this study. According to De Boever et al. [43], the correlation between MOE and density is strongly positive (*p* = 0.01). Hence, the density of wood can be used to predict the stiffness or MOE of LVL samples. Based on the result from Khoo et al. [1], the density distribution of small-diameter rubber trees was uniform. As the density was distributed uniformly within the rubber tree, and the fiber length of a small-diameter rubber tree does not seem to vary significantly, the effect of the glue spread rate on the MOE of rubberwood LVL samples in the flatwise direction was insignificant.

According to PRL-501 [26], the performance standard for laminated veneer lumber (LVL), the minimum MOR and MOE for LVL to be used in structural applications are 32.58 and 10,342.14 MPa, respectively. Based on this study, the MOR in the flatwise direction for rubberwood LVL produced using a 200 g/m^2^ glue spread rate is 91.05 MPa. This shows that the MOR in the flatwise directions has reached the minimum criterion (44.88 MPa) for the 2.1E-3100F stress class according to the performance standard for LVL (PRL-501) [26]. Additionally, the MOE in the flatwise direction for the rubberwood LVL produced using a 200 g/m^2^ glue spread rate was 11,189.55 MPa, which also reached the minimum requirement for the 1.5E-2250F stress class according to PRL-501 [26].

#### 3.3.2. Compressive Strength Parallel to the Longitudinal Axis

According to Table 4, the glue spread rate has highly significant effects (*p* ≤ 0.01) on the compressive strength parallel to the longitudinal axis of the rubberwood LVL. The compressive strength parallel to the longitudinal axis is 50.23, 48.04 and 46.93 MPa for the rubberwood LVL produced with 200, 240 and 280 g/m^2^ glue spread rates. The compressive strength parallel to the longitudinal axis of rubberwood LVL from this study met the minimum requirement for the 2.1E-3100F stress class according to PRL-501 [26]. The rubberwood LVL bonded using the lowest glue spread rate, which was 200 g/m^2^, had a significantly higher compression strength compared to those using higher glue spread rates. An increase in the glue spread rate to 240 g/m^2^ and 280 g/m^2^ reduced the compression strength by 4.4% and 6.6%, respectively.

Table 5 shows the failure modes from compression parallel to the longitudinal axis tested for rubberwood LVL bonded using 200, 240 and 280 g/m^2^ glue spread rates. Crushing failure occurs due to the weakness in compression and failure along the maximum compression line, whereas shear failure occurs on the maximum shear plane along 45°. Only two failure modes were observed in the samples—shear failure and crushing failure. The percentage of specimens with shear failure were 50%, 67% and 75% for rubberwood LVL bonded with 200, 240 and 280 g/m^2^ glue spread rates, respectively. The percentage of specimens with crushing failure were 50%, 33% and 25% for rubberwood LVL bonded with 200, 240 and 280 g/m^2^ glue spread rates, respectively. Compression specimens that failed with crushing showed no apparent delamination. Crushing failure was always associated with higher compressive properties due to the densification of the cell wall that collapsed in the crushing region, whereas shearing failure was due to the shearing between two fibers.

Total delamination was higher in specimens with higher glue spread rates, which greatly reduced the compression strength of the LVL. This also shows that higher glue spread rates contributed to higher failure at the gluebond layer. Glue spread rates of 240 and 280 g/m^2^ for young rubberwood veneers are excessive; the adhesive is squeezed out of the panel, the glueline thickness increases and, as a consequence, the adhesive strength decreases. It was stated by Bekhta et al. [44] that with the increasing of the glueline thickness, the bonding strength decreases and with a thicker glueline, higher internal stress is generated during glue shrinkage, which can lead to a lower shear strength. Moreover, at the high glue spread level, gas pressure increased significantly due to the high MC in the glueline, which generally led to blisters or blows, which could largely deteriorate the bonding quality of the laminated panel [45].

#### 3.3.3. Tensile Strength Parallel to the Longitudinal Axis

Based on Table 4, the effect of the glue spread rate on the tensile strength parallel to the longitudinal axis was not significant (*p* > 0.05). The average tensile strength parallel to the longitudinal axis was 50.38, 51.15 and 50.39 MPa for the rubberwood LVL produced with 200, 240 and 280 g/m^2^ glue spread rates. The tensile strength parallel to the longitudinal axis of rubberwood LVL from this study met the minimum requirements for the 2.1E-3100F stress class according to PRL-501 [26].

#### 3.3.4. Gluebond Shear Strength and Percentage of Wood Failure

**Figure 6:**
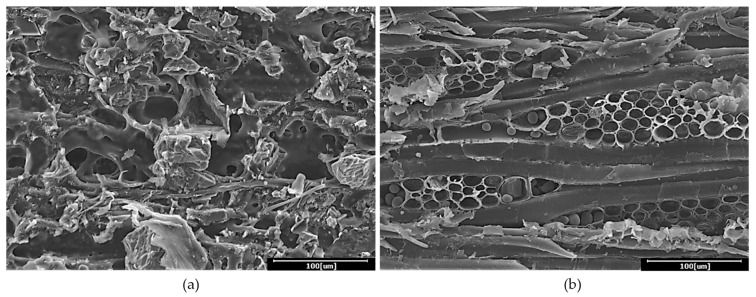
Veneer surface from delamination test observed using SEM with 500× magnification; (**a**) glueline failure and (**b**) wood failure.

The shear strength test is a common indicator of adhesive behavior in laminated panels. Gluebond shear strength was not significantly (*p* > 0.05) affected by the glue spread rate (Table 4); hence, the shear strength of specimens with thicker gluelines was equal to the shear strength of specimens with thin gluelines. However, percentages of wood failure were generally somewhat lower for the thicker gluelines than for thin gluelines. The mean percentages of wood failure of rubberwood LVL bonded with 200, 240 and 280 g/m^2^ glue spread rates were 83%, 70% and 38%, respectively. Rubberwood LVL produced using a 200 g/m^2^ glue spread rate showed the highest percentage of wood failure of 83.33% with the lowest coefficient of variance of 18.33%. When higher glue spread rates were used, the bonding strength between veneers increased until, at one point, additional adhesive does not contribute to the strength anymore [46]. The high failure in the glueline during the delamination test occurs because the value of the shear strength provided by the adhesive is lower than the value of the shear strength of the rubberwood veneer parallel to the grain in this study. Figure 6 shows the veneer surfaces from the delamination test due to glueline and wood failure. A major factor contributing to the higher rate of glueline failure from the use of higher glue spread rates was due to the formation of thick porous gluelines full of cavities and cracks, as shown in Figure 6a. The cavities and cracks were mainly generated during hot pressing in an effort to cure and impregnate the thick glueline into the voids in the veneer, which led to blisters or blows from the pressure generated. The higher the amount of glue applied, the higher the pressure generated, which led to the higher tendency of the panels to blister or blow in the hot press, which was observed in this study.

The large amount of starch granules in young rubberwood can limit the migration of the glueline, which resulted in a major reduction of the mechanical properties of the glue joint with the increase in the glue spread rate. The failure of wood in the delamination test exposed the ray cells of the rubberwood which were embedded with strach granules (Figure 6b). These results indicate that a glue spread rate of 200 g/m^2^ was the maximum amount of adhesive to be used in order to avoid failure along the glueline, while allowing bonding strength to be left unchecked in the structural design process. As reported by Kawalerczyk et al. [47], limiting the occurrence of microcracks in the bondlines is one of the key factors determining the strength properties of glue joints.

The bonding performance of ruberwood LVL produced using 200 g/m^2^ passed the minimum requirement for Voluntary Product Standard PS 2-04 for Structural Plywood [48]. According to Voluntary Product Standard PS 2-04, the average wood failure of specimens shall be not less than 80% [48]. Additionally, the highest gluebond shear strength of rubberwood LVL from this study—6.46 MPa—fulfilled the minimum shear test values of the 2.1E-3100F stress class for the PRL-50 performance standard for LVL [26].

## 4. Conclusions

The glue spread rate of 200 g/m^2^ was found to be adequate for the production of LVL produced with spindleless rotary veneers recovered from short-rotation *Hevea* plantation logs. The presence of starch granules in ray cells of young rubberwood trees limits the migration of the glueline, which leads to the creation of a thick porous glueline with an increase in the glue spread rate. Using the highest glue spread rate of 280 g/m^2^, we improved the physical properties of rubberwood LVL, which particularly contributed to higher board density and better dimensional stability. By contrast, rubberwood LVLs produced using a 200 g/m^2^ glue spread rate showed better mechanical properties, especially in the MOR in the flatwise direction and compressive strength parallel to the longitudinal axis. The mechanical properties of rubberwood LVL produced in this study met the minimum requirement for the 2.1E-3100F stress class, except for MOE in the flatwise direction. The MOE in the flatwise direction reached the minimum requirement for the 1.5E-2250F stress class. The application of phenol formaldehyde resin at more than 200 g/m^2^ was considered excessive, as a higher glue spread rate does not contribute to better mechanical properties in LVL.

## Figures and Tables

**Table 1:** Properties of spindleless rotary-peeled veneers with 2 mm thickness recovered from short-rotation *Hevea* plantation logs [1].

Lathe Check Properties		Contact Angle (°C) after 10 s
Depth (%)	Frequency per 5 cm	
50 ± 15	30 ± 10	6

**Table 2:** Cutting size of specimens for physical and mechanical tests.

Test	Size of Specimen (mm)	Number of Specimen Tested per Board
Moisture content	50 × 50 × 12	6
Density; specific gravity	50 × 50 × 12	6
Water absorption	50 × 50 × 12	6
Radial; tangential; longitudinal; volumetric swelling	50 × 50 × 12	6
Static bending in the flatwise direction	316 × 50 × 12	6
Compression parallel to the longitudinal axis	60 ×20 × 12	3
Tension parallel to the longitudinal axis	250 × 25 × 12	3
Gluebond shear test	81 × 25 × 12	3

**Table 3:** Mean values of physical properties of rubberwood LVL produced using different glue spread rate.

Physical Properties	Glue Spread Rate (g/m^2^)			Pr > F
	200	240	280	
Moisture content (%)	12.68 ^a^ (2.66)	12.91 ^a^ (4.92)	13.13 ^a^ (6.32)	n.s
Density (kg/m^3^)	864.11 ^a^ (2.44)	874.73 ^ab^ (2.82)	893.02 ^b^ (3.18)	**
Specific gravity	0.8261 ^a^ (2.66)	0.8611 ^b^ (2.56)	0.8793 ^c^ (2.42)	**
Water absorption after 2 h (%)	9.67 ^b^ (6.53)	9.14 ^b^ (8.89)	7.98 ^a^ (11.22)	**
Water absorption after 24 h (%)	28.56 ^b^ (3.89)	26.01 ^a^ (5.93)	25.48 ^a^ (5.46)	**
Radial swelling after 2 h (%)	2.59 ^b^ (16.21)	2.35 ^b^ (12.44)	2.00 ^a^ (16.52)	**
Radial swelling after 24 h (%)	4.93 ^b^ (11.98)	4.82 ^b^ (13.09)	4.00 ^a^ (16.39)	**
Tangential swelling after 2 h (%)	0.566 ^b^ (6.99)	0.570 ^b^ (10.39)	0.518 ^a^ (3.67)	**
Tangential swelling after 24 h (%)	1.342 ^b^ (10.21)	1.192 ^a^ (12.21)	1.154 ^a^ (12.71)	**
Longitudinal swelling after 2 h (%)	0.122 ^b^ (17.05)	0.090 ^a^ (19.62)	0.076 ^a^ (19.46)	**
Longitudinal swelling after 24 h (%)	0.146 ^b^ (13.27)	0.153 ^b^ (15.91)	0.118 ^a^ (18.67)	**
Volumetric swelling after 2 h (%)	3.51 ^b^ (12.47)	3.17 ^a^ (10.04)	2.88 ^a^ (13.86)	**
Volumetric swelling after 24 h (%)	6.72 ^b^ (15.10)	6.45 ^b^ (9.99)	5.44 ^a^ (14.57)	**

Note: Means followed by the same letters in the same row are not significantly different at *p* ≤ 0.05 according to Tukey’s test. Values in parentheses indicate coefficient of variance. n.s: not significant at *p* > 0.05; **: significant at *p* ≤ 0.01.

**Table 4:** Mean values of mechanical properties of rubberwood LVL produced using different glue spread rates.

Mechanical Properties	Glue Spread Rate (g/m^2^)			Pr > F
	200	240	280	
MOR in flatwise direction (MPa)	91.0518 ^a^ (5.15)	85.1048 ^b^ (1.53)	83.5965 ^b^ (5.44)	**
MOE in flatwise direction (MPa)	11189.55 ^a^ (10.04)	10767.51 ^a^ (5.99)	10278.66 ^a^ (6.88)	n.s
Compressive strength parallel to the longitudinal axis (MPa)	50.2340 ^a^ (5.74)	48.0382 ^b^ (4.38)	46.9340 ^b^ (4.47)	**
Tensile strength parallel to the longitudinal axis (MPa)	50.3791 ^a^ (5.17)	51.1458 ^a^ (6.11)	50.3899 ^a^ (2.43)	n.s
Gluebond shear strength (MPa)	6.46 ^a^ (4.64)	6.44 ^a^ (8.88)	5.84 ^a^ (12.33)	n.s

Note: Means followed by the same letters in the same row are not significantly different at *p* ≤ 0.05 according to Tukey’s test. Values in parentheses indicate coefficient of variance. n.s: not significant at *p* > 0.05; **: significant at *p* ≤ 0.01.

**Table 5:** Compression failure of rubberwood LVL bonded with different glue spread rates.

Glue Spread Rate (g/m^2^)	Compression Failure Characteristics	Percentage of Specimens with Failure Modes (%)		Percentage of Specimens with Total Delamination (%)
		Crushing	Shearing	
200	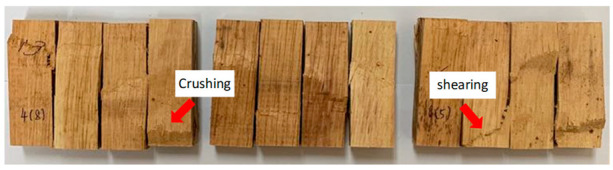	50	50	0
240	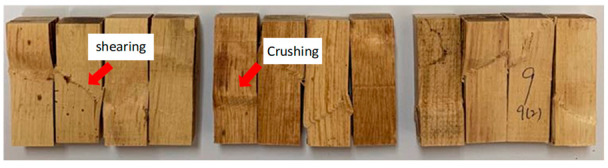	33	67	18
280	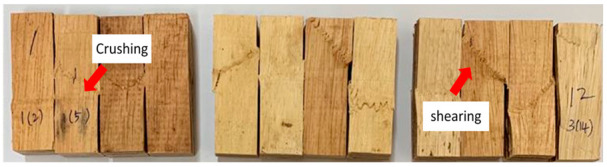	25	75	33

## Data Availability

All the data has been included in this paper. No other supporting data.

## References

[1] Khoo P.S., H’ng P.S., Chin K.L., Bakar E.S., Maminski M., Raja-Ahmad R.N., Lee C.L., Ashikin S.N., Saharudin M.H. (2018). Peeling of small diameter rubber log using spindleless lathe technology: Evaluation of veneer properties from outer to inner radial section of log at different veneer thicknesses. Eur. J. Wood Wood Prod..

[2] Ulker O., Rudawska A. (2016). Wood Adhesives and Bonding Theory. Adhesives—Applications and Properties.

[3] Frihart C.R., Rowell R.M. (2005). Wood Adhesion and Adhesives. Handbook of Wood Chemistry and Wood Composites.

[4] Kurt R., Cil M. (2012). Effects of press pressures on glueline thickness and properties of laminated veneer lumber glued with phenol formaldehyde adhesive. BioResources.

[5] Vick C.B. (1999). Adhesive Bonding of Wood Materials. Wood Handbook: Wood as an Engineering Material.

[6] Daoui A., Descamps C., Marchal R., Zerizer A. (2011). Influence of veneer quality on beech LVL mechanical properties. Maderas Cienc. y Tecnol..

[7] Loh Y.F., Paridah M.T., Yeoh B.H. (2011). Density distribution of oil palm stem veneer and its influence on plywood mechanical properties. J. Appl. Sci..

[8] Frihart C.R., Hunt C.G., Centennial (2010). Adhesives with Wood Materials—Bond Formation and Performance. Wood Handbook: Wood as an Engineering Material.

[9] Mohd Yusof N., Md Tahir P., Muhammad Roseley A.S., Lee S.H., Abdul Halip J., Mohammad Suffian James R., Ashaari Z. (2019). Bond integrity of cross laminated timber from Acacia mangium wood as affected by adhesive types, pressing pressures and loading direction. Int. J. Adhes. Adhes..

[10] Darmawan W., Nandika D., Massijaya Y., Kabe A., Rahayu I., Denaud L., Ozarska B. (2015). Lathe check characteristics of fast growing sengon veneers and their effect on LVL glue-bond and bending strength. J. Mater. Process. Technol..

[11] Khoo P.S., Chin K.L., H’ng P.S., Lee C.L., Bakar E.S., Ashaari Z., Abdullah L.C., Gandaseca S. (2020). Laminated veneer lumber from spindleless rotary-peeled veneers produced from short rotation, small hevea plantation logs: Effects of lamination pressure. BioResources.

[12] (2007). ASTM D4442-07: Standard Test Methods for Direct Moisture Content Measurement of Wood and Wood-Base Materials.

[13] (2014). ASTM D2395-14: Standard Test Methods for Specific Gravity of Wood-based Materials.

[14] (2012). ASTM D1037-12: Standard Test Methods for Evaluating Properties of Wood-Based Fiber and Particle Panel Materials.

[15] (2010). ASTM D5456-10: Standard Test Methods for Evaluation of Structural Composite Lumber Products.

[16] (2011). ASTM D906-11: Standard Test Method for Strength Properties of Adhesives in Plywood Type Construction in Shear by Tension Loading.

[17] Pizzi A., Mittal K.L., Pizzi A., Mittal K.L. (2017). Handbook of Adhesive Technology.

[18] Wang X., Huang L., Zhang C., Deng Y., Xie P., Liu L., Cheng J. (2020). Research advances in chemical modifications of starch for hydrophobicity and its applications: A review. Carbohydr. Polym..

[19] Din Z., Chen L., Xiong H., Wang Z., Ullah I., Lei W., Shi D., Alam M., Ullah H., Khan S.A. (2020). Starch: An Undisputed Potential Candidate and Sustainable Resource for the Development of Wood Adhesive. Starch/Staerke.

[20] Teoh Y.P., Don M.M., Ujang S. (2011). Assessment of the properties, utilization, and preservation of rubberwood (Hevea brasiliensis): A case study in Malaysia. J. Wood Sci..

[21] Wei B., Sun B., Zhang B., Long J., Chen L., Tian Y. (2016). Synthesis, characterization and hydrophobicity of silylated starch nanocrystal. Carbohydr. Polym..

[22] Jiang S., Dai L., Qin Y., Xiong L., Sun Q. (2016). Preparation and characterization of octenyl succinic anhydride modified taro starch nanoparticles. PLoS ONE.

[23] Ahmed W., Sagir M., Tahir M.S., Ullah S., Charminé H. (2019). Phenol Formaldehyde Resin for Hydrophilic Cellulose Paper. Advances in Sustainable and Environmental Hydrology, Hydrogeology, Hydrochemistry and Water Resources.

[24] De Oliveira R.G.E., Gonçalves F.G., de Segundinho P.G.A., da Oliveira J.T.S., Paes J.B., Chaves I.L.S., Brito A.S. (2020). Analysis of glueline and correlations between density and anatomical characteristics of Eucalyptus grandis x Eucalyptus urophylla glulam. Maderas Cienc. Tecnol..

[25] Tienne D.L.C., Nascimento A.M., Garcia R.A., Silva D.B. (2011). Adhesion quality of pine wood glued joints under internal and external service conditions. Floresta Ambient..

[26] APA (2000). PRL-501: Performance Standard for Laminated Veneer Lumber.

[27] Sulastiningsih I.M., Trisatya D.R., Balfas J. (2020). Some properties of laminated veneer lumber manufactured from oil palm trunk. IOP Conf. Ser. Mater. Sci. Eng..

[28] Abdul Khalil H.P.S., Nurul Fazita M.R., Bhat A.H., Jawaid M., Nik Fuad N.A. (2010). Development and material properties of new hybrid plywood from oil palm biomass. Mater. Des..

[29] Hashim R., Sarmin S.N., Sulaiman O., Yusof L.H.M. (2011). Effects of cold setting adhesives on properties of laminated veneer lumber from oil palm trunks in comparison with rubberwood. Eur. J. Wood Wood Prod..

[30] Sulaiman O., Salim N., Hashim R., Yusof L.H.M., Razak W., Yunus N.Y.M., Hashim W.S., Azmy M.H. (2009). Evaluation on the suitability of some adhesives for laminated veneer lumber from oil palm trunks. Mater. Des..

[31] He M., Xu D., Li C., Ma Y., Dai X., Pan X., Fan J., He Z., Gui S., Dong X. (2020). Cell wall bulking by maleic anhydride for wood durability improvement. Forests.

[32] Wang X., Deng Y., Li Y., Kjoller K., Roy A., Wang S. (2016). In situ identification of the molecular-scale interactions of phenol-formaldehyde resin and wood cell walls using infrared nanospectroscopy. RSC Adv..

[33] Furuno T., Imamura Y., Kajita H. (2004). The modification of wood by treatment with low molecular weight phenol-formaldehyde resin: A properties enhancement with neutralized phenolic-resin and resin penetration into wood cell walls. Wood Sci. Technol..

[34] Khalil H.P.S.A., Alwani M.S., Ridzuan R., Kamarudin H., Khairul A., Khalil H.P.S.A., Alwani M.S., Ridzuan R., Kamarudin H., Khairul A. (2008). Chemical Composition, Morphological Characteristics, and Cell Wall Structure of Malaysian Oil Palm Fibers. Polym. Plast. Technol. Eng..

[35] Khalid I., Sulaiman O., Hashim R., Razak W., Jumhuri N., Rasat M.S.M. (2015). Evaluation on layering effects and adhesive rates of laminated compressed composite panels made from oil palm (Elaeis guineensis) fronds. Mater. Des..

[36] Shukla S.R., Kamdem D.P. (2009). Properties of laboratory made yellow poplar (Liriodendron tulipifera) laminated veneer lumber: Effect of the adhesives. Eur. J. Wood Wood Prod..

[37] Rowell R.M., Youngs R.L. (1981). Dimensional Stabilization of Wood in Use.

[38] Augustina S., Wahyudi I., Darmawan I.W., Malik J., Basri E., Kojima Y. (2020). Specific gravity and dimensional stability of boron-densified wood on three lesser-used species from Indonesia. J. Korean Wood Sci. Technol..

[39] Chai L.Y., H’Ng P.S., Lim C.G., Chin K.L., Jusoh M.Z., Bakar E.S. (2011). Production of oil palm trunk core board with wood veneer lamination. J. Oil Palm Res..

[40] Nuryawan A., Abdullah C.K., Hazwan C.M., Olaiya N.G., Yahya E.B., Risnasari I., Masruchin N., Baharudin M.S., Khalid H., Abdul Khalil H.P.S. (2020). Enhancement of Oil Palm Waste Nanoparticles on the Properties and Characterization of Hybrid. Polymers.

[41] Khoo P.S., Chin K.L., Hng P.S., Bakar E.S., Lee C.L., Go W.Z., Dahali R. (2019). Physical properties and bonding quality of laminated veneer lumber produced with veneers peeled from small-diameter rubberwood logs. R. Soc. Open Sci..

[42] Li D., Hu X., Huang Z., Chen Y., Han H., Xiao C. (2018). Effect of several modifiers on the mechanical and tribological properties of phenol formaldehyde resin. High Perform. Polym..

[43] De Boever L., Vansteenkiste D., Van Acker J., Stevens M. (2007). End-use related physical and mechanical properties of selected fast-growing poplar hybrids (Populus trichocarpa x P. deltoides). Ann. For. Sci..

[44] Bekhta P., Sedliačik J., Bekhta N. (2020). Effects of selected parameters on the bonding quality and temperature evolution inside plywood during pressing. Polymers.

[45] Wang B.J., Dai C. (2005). Hot-pressing stress graded aspen veneer for laminated veneer lumber (LVL). Holzforschung.

[46] López L.F., Correal J.F. (2009). Exploratory study of the glued laminated bamboo Guadua angustifolia as a structural material. Maderas Cienc. Technol..

[47] Kawalerczyk J., Dziurka D., Mirski R., Trociński A. (2019). Flour fillers with urea-formaldehyde resin in plywood. BioResources.

[48] NIST (2004). PS 2-04: Performance Standard for Wood-Based Structural-Use Panels.

